# Mindfulness Training for Healthy Aging: Impact on Attention, Well-Being, and Inflammation

**DOI:** 10.3389/fnagi.2017.00011

**Published:** 2017-02-03

**Authors:** Stephanie Fountain-Zaragoza, Ruchika Shaurya Prakash

**Affiliations:** Clinical Neuroscience Laboratory, Department of Psychology, Ohio State UniversityColumbus, OH, USA

**Keywords:** mindfulness training, healthy aging, attentional control, psychological well-being, systemic inflammation

## Abstract

The growing interest in mindfulness interventions for use in aging samples has been met with promising evidence of cognitive, emotional, and physiological benefits. The purpose of this review is to provide an overview of the impact of mindfulness training on three areas of functioning in older adults: behavioral and neural correlates of attentional performance, psychological well-being, and systemic inflammation. We have previously proposed that mindfulness training is uniquely suited as a rehabilitative tool for conferring both cognitive and emotional benefits for older adults. Specifically, mindfulness training's promotion of focused attention may mitigate the decline of attentional control abilities across late development and allow older adults to capitalize on their preserved emotion regulation abilities. Existing evidence points to some improvements in facets of attentional control in older adults, although some studies have shown no benefits in performance. Further, there is evidence of enhancements in both psychological and physical aspects of well-being, and accompanying improvements in systemic inflammation, following mindfulness training. The scientific investigation of mindfulness training is still relatively nascent, with only a limited number of studies, particularly randomized controlled trials utilizing active comparison conditions. It will be important for future research to incorporate placebo-controlled comparison groups to clearly establish the causal role of mindfulness practices in promoting holistic health in older adults.

## Introduction

Mindfulness training has gained increasing traction in recent years as a feasible and promising intervention for enhancing facets of both psychological and physical health across development. Broadly defined as the cultivation of sustained attention in a framework of non-reactivity and acceptance (Kabat-Zinn, 1982), mindfulness training involves direction of attention to either one or multiple phenomena as they arise. These techniques often fall into three component types: (1) focused attention meditation involves sustained attention to a single object while monitoring for and disengaging from distractions (Lutz et al., 2008); (2) open monitoring meditation involves attending to the detailed features of transient phenomena without selective focus on one object (Lutz et al., 2008); (3) loving-kindness meditation involves cultivation of a universal state of love and compassion toward oneself and others (Salzberg, 2002). Thus, engagement in mindfulness practices requires the utilization of either narrowly focused attention (e.g., breath awareness or body scan practices) or broadly receptive attention (e.g., choiceless awareness or gratitude practices).

Training in such mindfulness practices has been evaluated for its prophylaxis for various metrics of overall health including, but not limited to, improvements in behavioral and neural metrics of cognitive functioning, particularly attentional control (Tang et al., 2015); regulation of affective experiences (Chiesa et al., 2013); reductions in overall levels of perceived stress and systemic inflammation (Creswell et al., 2012; Rosenkranz et al., 2013); and improvements in overall well-being and psychological health (Baer, 2003). Although the majority of these studies have been conducted in young adults and community participants, there is a growing interest in the application of mindfulness training as a preventative intervention targeting the elderly. This population is of particular interest given the age-related declines in social support, limitations to physical independence, and decrements in several domains of cognitive function.

**Figure 1:**
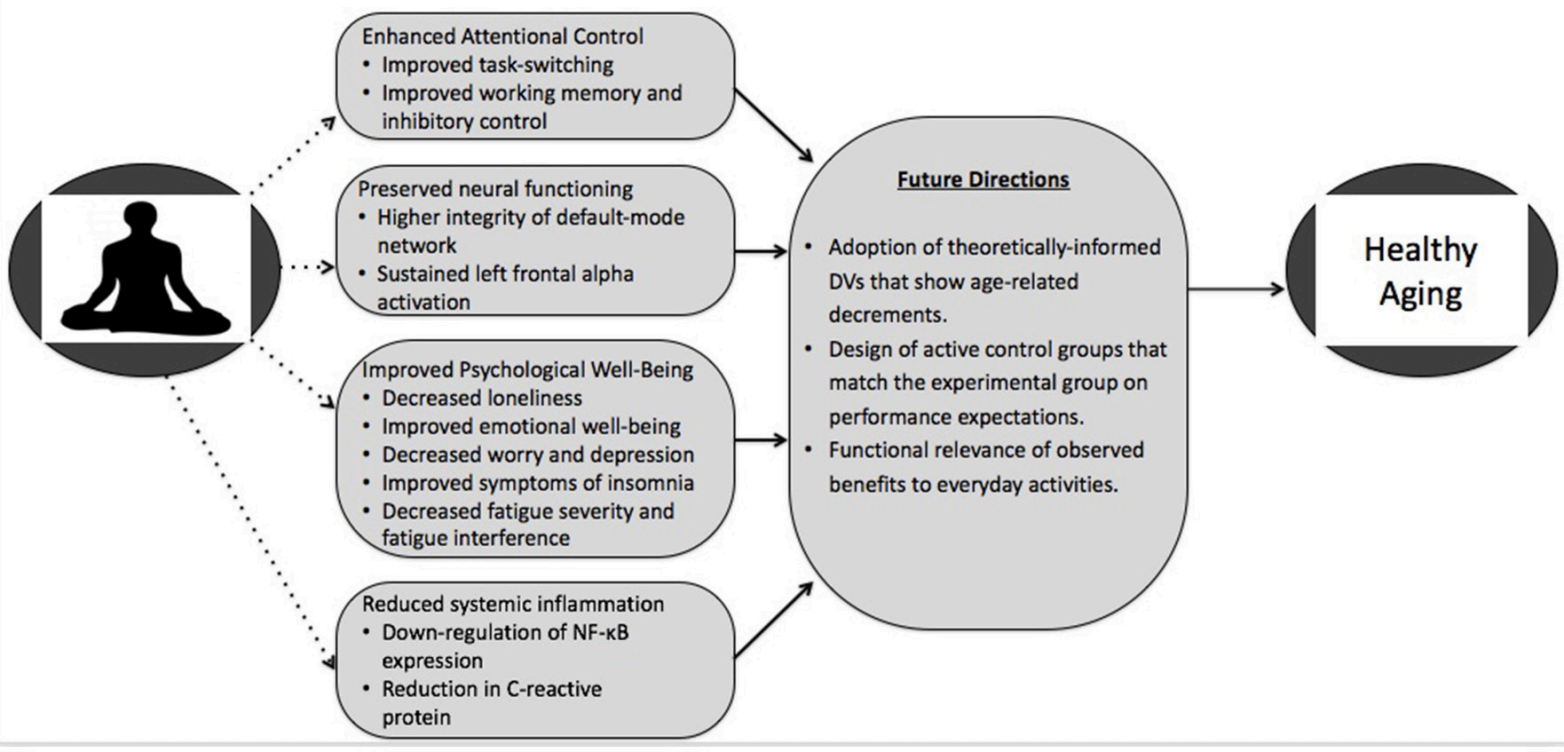
**An illustration of the known and examined potential mechanisms through which mindfulness training may exert its influence on healthy aging (dotted lines demonstrating a tentative link between mindfulness and various pathways)**. Given the infancy of this research and the lack of rigorous RCT designs, the evidence linking mindfulness training to improvements in attentional control, psychological well-being, and systemic inflammation, though promising, needs more conclusive support. Incorporation of the suggested future directions will aid in providing a more concrete, definitive test (represented by solid lines) for the efficacy of mindfulness training in promoting healthy aging.

We have previously proposed that mindfulness training is particularly useful in aging populations as it orients the practitioner, in an accepting and non-judgmental framework, to the mind's tendency to wander (Bishop et al., 2004), thereby promoting the use of attentional control and allowing older adults to capitalize on the preserved emotion regulation abilities that are observed with aging (Prakash et al., 2014). As a follow-up to this proposed paradigm recommending mindfulness training as a rehabilitative tool for conferring emotion-cognition benefits in older adults, this review provides a critical summary of the recent literature examining the impact of mindfulness training for behavioral and neural correlates of attentional control. One of Dr. Raja Parasuraman's seminal contributions to the literature, in collaboration with his colleagues, is the nuanced examination of age-related differences in the shifting, scaling, and maintenance of attentional control. This work detailing older adults' decrements in these areas, provides a theoretically-informed set of outcome variables for use in this population. Although there is some evidence that mindfulness yields benefits for attentional abilities with age, we believe future research examining the potential of mindfulness training in mitigating age-related attentional decrements can benefit tremendously from implementing measures derived from Dr. Parasuraman and colleagues' key findings. Further, given that successful aging is truly a metamorphosis of cognitive, affective, and physiological health, we go beyond reviewing the cognitive potential of mindfulness training to include a brief review of the existent literature examining alterations in psychological well-being and systemic inflammation resulting from mindfulness training in older adults (See Figure 1). An electronic search was conducted in *PubMed, PsychInfo*, and *Web of Science* using the keywords *mindfulness, older adults, aging, attentional control, cognition, well-being*, and *inflammation*. We then inspected the reference sections of all retrieved articles for a cross-reference. We included articles written in English that were published prior to September, 2016. Please see Table 1 for a list of the reviewed studies and a brief summary of their findings.

**Table 1:** **Brief summaries of reviewed studies categorized by outcome (attentional control, psychological well-being, and inflammatory processes)**.

**References**	**Titles**	**Study type**	**Population**	**Intervention group**	**Comparison group**	**At-home practice**	**Dependent variables**	**Main findings**
**ATTENTIONAL CONTROL: BEHAVIORAL AND NEURAL FUNCTIONING**								
Prakash et al., 2012b	Mindfulness disposition and default-mode network connectivity in older adults	Correlational	Older adults (*n* = 25, mean age = 66)	n/a	n/a	n/a	Resting-state default-mode network connectivity	Trait mindfulness is associated with greater connectivity in the dorsal posterior cingulate cortex and precuneus, regions of the default-mode network.
Fiocco and Mallya, 2015	The importance of cultivating mindfulness for cognitive and emotional well-being in late life	Correlational	Older adults (*n* = 73, mean age = 69)	n/a	none	n/a	Set-shifting (trail making test A & B), verbal fluency (controlled oral word association task), declarative memory (california verbal learning test).	Trait mindfulness is positively associated with set-shifting, but not verbal fluency or declarative memory.
Prakash et al., 2015	The role of emotion regulation and cognitive control in the association between mindfulness disposition and stress	Correlational	Older adults (*n* = 48, mean age = 65)	n/a	Young adults (*n* = 50, mean age = 24)	n/a	Inhibitory control (flanker task), working memory (N-back task), set-shifting (Task-switching paradigm)	Trait mindfulness is not significantly associated with inhibitory control, working memory, or set-shifting in older adults.
Pagnoni and Cekic, 2007	Age effects on gray matter volume and attentional performance in zen meditation	Cross-sectional comparison	Adult meditators (*n* = 13, mean age = 37, meditation experience = >3 years daily)	n/a	Older-adult non-meditators (*n* = 13 mean age = 36)	n/a	Sustained attention (rapid visual information processing task)	Meditators did not show a significant negative correlation between gray matter volume and attentional performance with age, as observed in controls.
van Leeuwen et al., 2009	Age effects on attentional blink performance in meditation	Cross-sectional comparison	Older-adult meditators (*n* = 17, mean age = 50, meditation experience = 1-20 years)	n/a	Older-adult non-meditators (*n* = 17, mean age = 50), young-adult non-meditators (*n* = 17, mean age = 24)	n/a	Temporal capacity of attention (attentional blink task)	Older-adult meditators showed a smaller attentional blink than age-matched and young-adult non-meditators.
Prakash et al., 2012a	Long-term concentrative meditation and cognitive performance among older adults	Cross-sectional comparison	Older-adult meditators (*n* = 20, mean age = 59, meditation experience = >10 years)	n/a	Older-adult non-meditators (*n* = 20, mean age = 60)	n/a	Working memory (digit span), response inhibition (stroop color word task), processing speed (letter cancelation, digit symbol substitution), set-shifting Ttrail making test, rule shift card test	Older-adult meditators performed better than age-matched non-meditators on all tests of attention except the digit span backwards test.
Lenze et al., 2014	Mindfulness-based stress reduction for older adults with worry symptoms and co-occuring cognitive dysfunction	Feasbility	Older adults with clinically significnat anxiety and cognitive dysfunction (*n* = 34, mean age = 71)	8-week MBSR: 2.5-h weekly meetings and a 1-day retreat (*n* = 16, mean age = 71)	12-week MBSR: 2.5-h weekly meetings and a 2.5-h retreat day (*n* = 18, mean age = 71)	Not explicitly stated, but followed the MBSR protocol that presumably included at-home practices	Verbal fluency, response inhibition (stroop task), short-term working memory (digit span forward), list learning (immediate and delayed), paragraph learning (immediate and delayed),	Significant pre to post changes were observed for list learning (delayed recall), paragraph learning (immediate and delayed), verbal fluency, and response inhibition.
O'Connor et al., 2014	The effects of mindfulness-based cognitive therapy on depressive symptoms in elderly bereaved people with loss-related distress: a controlled pilot study	Feasibility	Distressed older adults (*n* = 65, mean age = 78)	MBCT: 2-h weekly meetings for 8 weeks (*n* = 32, mean age = 78)	Wait-list group (*n* = 33, mean age = 77)	40 min of daily practice	Working memory (letter-number sequencing from WAIS-III administered via telephone)	Mindfulness group showed improvements in working memoyt at post-training compared to wait-list control participants.
McHugh et al., 2010	Mindfulness as a potential intervention for stimulus over-selectivity in older adults	Experimental induction	Older adults (*n* = 24, mean age = 79)	10-min mindfulness induction	10-min unfocused attention" induction	n/a	stimulus over-selectivity	Brief mindfulness induction significantly reduced stimulus over-selectivity
Mallya and Fiocco, 2016	Effects of mindfulness training on cognitive and well-being in older adults	Quasi-RCT	Older adults (*n* = 97, mean age = 69)	MBSR: 2.5-h weekly meetings for 8 weeks (*n* = 57)	Reading and Relaxation group: 2.5-h weekly meetings for 8 weeks (*n* = 40)	30 min. of daily practice	Simple attention and switching (trail making tests A and B), processing speed (controlled oral word association test), episodic memory (CVLT)	No significant differences between groups on any of the measures following the intervention.
Alexander et al., 1989	Transcendental meditation, mindfulness, and longevity: an experimental study with the elderly	RCT	Older adults (*n* = 62, mean age = 81 years)	30 min. once/week sessions for 8 weeks: 1) Transcendental Meditation (*n* = 20); 2) Langer method of mindfulness meditation (*n* = 21)	Relaxation group (*n* = 21); no treatment (*n* = 11)	20 min of twice daily practice for the active groups	Paired associate learning (DST subtest), cognitive flexibility (overlearned verbal task, Stroop color word test [RT interference]), perceived control, verbal fluency (DST subtest)	TM and mindfulness groups improved more than both control groups on paired associate learning and one measure of cognitive flexibility. TM outperformed mindfulness meditation on both of these measures.
Moynihan et al., 2013	Mindfulness-based stress reduction in older adults: effects on executive function, frontal alpha asymmetry and immune function.	RCT	Older adults (*n* = 201, mean age = 73.4)	MBSR: 2.5-h weekly meetings for 8 weeks and a 7-h intensive retreat (*n* = 101; mean age = 73),	Wait-list group (*n* = 100, mean age = 74)	none	Processing speed (trail making test A) and set-shifting (trail making B/A ratio); left frontal alpha asymmetry	There were improvements in set-shifting and a reduced shift to rightward frontal alpha activation immediately following mindfulness training compared to wait-list, but these effects were not maintained at follow-up.
**PSYCHOLOGICAL WELL-BEING**								
Fiocco and Mallya, 2015	The importance of cultivating mindfulness for cognitive and emotional well-being in late life	Correlational	Older adults (*n* = 73, mean age = 69)	n/a	none	n/a	Psychological well-being (depressive symptoms, quality of life, stress)	Trait mindfulness is associated with greater psychological well-being on all measures.
Prakash et al., 2015	The role of emotion regulation and cognitive control in the association between mindfulness disposition and stress	Correlational	Older adults (*n* = 48, mean age = 65)	n/a	Young adults (*n* = 50, mean age = 24)	n/a	Perceived stress, emotion dysregulation	Trait mindfulness is negatively associated with perceived stress and emotion dysregulation mediates this relationship.
Prakash et al., 2017	Mindfulness and emotion regulation in older and young adults	Correlational	Older adults (*n* = 48, mean age = 65)	n/a	Young adults (*n* = 49, mean age = 24)	n/a	Emotion dysregulation, emotion regulation strategy use	Thought avoidance mediates the association between trait mindfulness and emotion dysregulation across both age groups. Age moderated this effect such that less mindfulness in young adults is associated with greater use of thought avoidance and greater emotion dysregulation.
Morone et al., 2008	“I felt like a new person.” The effects of mindfulness meditation on older adults with chronic pain: qualitative narrative analysis of diary entries	Feasability (qualitative)	Older adults with chronic pain (*n* = 27, mean age = 74)	8-week mindfulness meditation program modeled on MBSR: 1.5-h weekly meetings	none	50 min of daily practice	Diary contents	Diary themes reflected beneficial effects on pain, attention, sleep (latency and quality), achieving well-being (mood elevation, global quality of life).
Splevins et al., 2009	Do improvements in emotional distress correlate with becoming more mindful? A study of older adults	Feasibility	Older adults with significant stress or symptoms of depression or anxiety (*n* = 22, mean age = 65 years)	MBCT: 2-h weekly meetings for 8 weeks	none	none	Emotional wellbeing (depression, anxiety, stress levels)	Signfiicant improvements in emotional well-being post-MBCT. Increased mindfulness associated with improved emotional wellbeing; act with awareness and accept without judgmenet associated with reduced depression.
Szanton et al., 2011	Examining mindfulness-based stress reduction: perceptions from minority older adults residing in a low-income housing facility	Feasibility (qualitative focus groups)	Older-adult, African American, low-income women (*n* = age range = 60-90)	Mindfulness meditation program modeled on MBSR: meeting duration not specified	none	none	Focus group discussion content	Primary themes: stress management, applying mindfulness, social support of group meditation; Used MBSR for stressors: growing older with physical pain, medical tests, financial strain, having grandchildren with significant mental, physical, financial, or legal hardships; used MBSR for coping with medical procedures and managing depression and anger.
Foulk et al., 2014	Mindfulness-based cognitive therapy with older adults: an exploratory study	Feasibility	Older adults with depression and/or anxiety (*n* = 50, mean age = 73)	MBCT: weekly meetings for 8 weeks and one 6-h retreat	none	30-40 min of daily practice	Anxiety (hospital anxiety and depression scale), rumination (ruminative responses scale), depressive symptoms (geriatric depression scale- short form), insomnia (sleep problems scale)	Participation in MBCT resulted in significant improvements in reported anxiety, ruminative thoughts, and sleep problems; reduced depressive symptoms.
Morone et al., 2009	A mind–body program for older adults with chronic low back pain: results of a pilot study	RCT	Older adults with lower back pain (*n* = 40, mean age = 78)	8-week mindfulness meditation program modeled on MBSR: 1.5-h weekly meetings	Education group (*n* = 20, mean age = 73)	50 min of daily practice	Disability, psychological function, pain severity	Both groups improved on measures of disability, pain, and psychological function at post-intervention and 4-month follow-up. No between group differences.
Young and Baime, 2010	Mindfulness-based stress reduction: effect on emotional distress in older adults	Feasibility	Older adults with clinically significant depression and anxiety (*n* = 141, mean age = 65)	MBSR: weekly meetings for 8 weeks	none	45 min of daily practice	Change in mood states (profile of mood states, short form)	Overall emotional distress and all subscales improved signfiicantly, >50% reduction in number of older adults with clinically significant depression and anxiety
Creswell et al., 2012	Mindfulness-based stress reduction training reduces loneliness and pro-inflammatory gene expression in older adults: a small randomized Controlled Trial	RCT	Older adults (*n* = 40, mean age = 65 years)	MBSR: 2-h weekly meetings for 8 weeks and one 7-h retreat (*n* = 20, mean age = 64)	Wait-list group (*n* = 20, mean age = 65)	30 min of daily practice	Loneliness	Mindfulness training produced greater reductions in loneliness than the wait-list group.
Lenze et al., 2014	Mindfulness-based stress reduction for older adults with worry symptoms and co-occuring cognitive dysfunction	Feasbility	Older adults with clinically significnat anxiety and cognitive dysfunction (*n* = 34, mean age = 71)	8-week MBSR: 2.5-h weekly meetings and a 1-day retreat (*n* = 16, mean age = 71)	12-week MBSR: 2.5-h weekly meetings and a 2.5-h retreat day (*n* = 18, mean age = 71)	Not explicitly stated, but MBSR protocol includes at-home practices	Worry (Penn State worry questionaire)	Participants exhibited improvements in worry severity, with no additional benefit of the 12-week intervention over the 8-week intervention.
**INFLAMMATORY PROCESSES**								
Creswell et al., 2012	Mindfulness-based stress reduction training reduces loneliness and pro-inflammatory gene expression in older adults: a small randomized controlled trial	RCT	Older adults (*n* = 40, mean age = 65 years)	MBSR: 2-h weekly meetings for 8 weeks and one 7-h retreat (*n* = 20, mean age = 64)	Wait-list group (*n* = 20, mean age = 65)	30 min of daily practice	Pro-inflammatory gene expression (NF-κB leukocytes, C reactive protein)	Baseline loneliness was associated with and NF-κB gene expression. Compared to the wait-list group, MBSR participants exhibited down-regulation of NF-κB expression and reduced C-reactive protein levels, but no difference in IL-6 levels.
Gallegos et al., 2013	Toward identifying the effects of the specific components of mindfulness-based stress reduction on biologic and emotional outcomes among older adults	RCT	Older adults (*n* = 200)	MBSR (*n* = 100, mean age = 72)	Wait-list group (*n* = 100) not included in analysis	Participants engaged in at-home activities, but requirement not specified	Immune function (IL-6), circulating insulin-like growth factor (IGF)-1 concentrations, positive affect	More yoga practice associated with higher post-treatment IGF-1 and greater improvement in positive affect across intervention; sitting meditation associated with post-treatment IGF-1; greater use of body scanning associated with reduced antigen-specific IgM and IgG 3 weeks postintervention (but not 24 weeks); no associations between MBSR activities and IL-6 levels.
Moynihan et al., 2013	Mindfulness-based stress reduction in older adults: effects on executive function, frontal alpha asymmetry and immune function.	RCT	Older adults (*n* = 201, mean age = 73.4)	MBSR: 2.5-h weekly meetings for 8 weeks and a 7-h intensive retreat (*n* = 101; mean age = 73),	Wait-list group (*n* = 100, mean age = 74)	none	Antibody response (immunoglobulin G response to protein antigen).	There were higher baseline antibody levels after MBSR, but lower antibody responses 24 weeks after antigen challenge.
Black et al., 2015	Mindfulness meditation and improvement in sleep quality and daytime impairment among older adults with sleep disturbances	RCT	Older adults with moderate sleep disturbance (*n* = 49, mean age = 66)	Mindful awareness practices intervention: 2-h weekly meetings (*n* = 24, mean age = 67)	Sleep hygeine education (*n* = 25, mean age = 66)	Graduated daily practice (5-20 min)	Sleep disturbance (pittsburgh sleep quality index); insomnia, depression, anxiety, stress, and fatigue; inflammatory signaling NF-κB	Mindfulness group improved more on PSQI, insomnia symptoms, depression symptoms, fatigue interference, fatigue severity. No between group differences for anxiety, stress, or NF-κB (but these levels declines significantly over time in both groups).

## Mindfulness and behavioral correlates of attentional control

### Age-related alterations in attentional control

Attentional control is broadly defined as the ability to streamline information processing by selecting and amplifying task-relevant information while ignoring irrelevant, interfering information in order to conduct complex goal-oriented behaviors (Petersen and Posner, 2012). Attentional control thus encompasses a wide variety of cognitive processes ranging from those that operate upon information arising from external stimuli, such as orienting and discriminating, to those that require the use of internal information stored in working memory and long-term memory or that are dictated by internal task-sets (Chun et al., 2011). There is an extensive literature documenting age-related changes in attentional control processes, with aggregate evidence that older adults exhibit deficits on many tasks of attentional control (Hasher and Zacks, 1988; Parasuraman and Greenwood, 1998; Braver and West, 2008; Lustig and Jantz, 2015). The work of Dr. Raja Parasuraman and colleagues contributed substantially to our understanding of the deficits in various facets of attention that come with age. Greenwood and Parasuraman (1999) developed a two-component model of visual search that includes shifting and scaling, making use of cued-visual search tasks in order to elucidate age-related changes in the allocation of attention. These studies showed age-related decrements in voluntarily shifting of visuospatial attention from one hemifield to another following invalid location cues (Greenwood et al., 1993), as well as poorer attentional disengagement following invalid cues (Greenwood and Parasuraman, 1994). When the scale of spatial attention was systematically altered by the presentation of location precues of varying sizes (i.e., cueing a single letter, a column of letters, or the entire array), the ability to effectively adjust the focus of attention declined with age and was lowest for those over the age of 75 (Greenwood et al., 1997). Subsequent work showed that the performance benefits of valid precues observed in younger adults initially increased with age (ages 65–74), but then decreased with advanced age (ages 75–85), providing evidence that aging is associated with a decreasingly focused attentional beam (Greenwood and Parasuraman, 2004). Both the shifting and scaling of attentional focus, occurring very early in information processing, might be augmented by mindfulness training as practitioners develop more acute attention to present-moment experiences.

In addition to age-related declines in selective attention, age decrements in sustained attention have also been systematically evaluated. Employing vigilance tasks in which participants are asked to respond to targets and withhold responses to non-targets, older adults were found to exhibit decreased hit rates and increased false-alarm rates compared to young adults (Parasuraman and Giambra, 1991; Filley and Cullum, 1994), and these differences did not disappear with increased practice in older adults (Parasuraman and Giambra, 1991). Vigilance may also be parceled into two types, sensory and cognitive, that can be evaluated by presenting participants with pairs of digits and instructing them to discriminate between the physical size (sensory vigilance) or the numeric value of the digits (cognitive vigilance). Evaluations of these two conditions provided evidence that older adults exhibited lower detection rates than young adults on both task types, but that false alarm rates were greater in older adults, particularly in the sensory task (Deaton and Parasuraman, 1993). This suggests that older adults might experience significant declines in perceptual processes, which might relate to the deficits in visual selective attention outlined above. Work employing signal detection indices, which incorporate both hits and false alarms, has suggested equivalent overall vigilance across age groups for tasks requiring both automatic and effortful stimulus processing and no differences in sustained attention decrements over time (Berardi et al., 2001). Interestingly, older adults changed the strategy used for target detection during more difficult conditions such that they limited attention to one feature of the target, leading to decreases in overall sensitivity compared to young adults, but preventing vigilance decrements at higher demands.

There is relatively strong support for age-related declines in executive types of attention (e.g., Chao and Knight, 1997; Andrés and Van der Linden, 2000; Milham et al., 2002; Davidson et al., 2003a). Although a set of meta-analyses found limited age-related deficits in local task-switching and several selective attention tasks, including inhibition, negative priming, flanker, and Stroop tasks (Verhaeghen, 2011), there was evidence of age-related differences in tasks of divided attention, including dual tasking and global task-switching (Verhaeghen, 2011; Wasylyshyn et al., 2011). It is important to note that sustained and executive attentions rely in part on the efficient and accurate allocation of attention. Thus, all of these facets of attentional control might be meaningfully impacted by the focused attention practices incorporated in mindfulness training, allowing for the sharpening of attentional focus and conscious maintenance of goal-directed attention.

### Mindfulness and facets of attentional control

Given that attentional control is posited to be a primary skill that is utilized during and facilitated by engagement in mindfulness practices, and considering the reviewed evidence that attentional control abilities decline with age, there is an emerging literature examining the impact of mindfulness on attentional control abilities in older adults. Within the mindfulness and attention literature, researchers have primarily employed three study designs: (1) correlational studies examining associations between trait levels of mindfulness and performance on attentional tasks, (2) cross-sectional comparisons of individuals with extensive mindfulness experience (i.e., expert meditators) and meditation-naïve individuals on attentional tasks, and (3) longitudinal studies of change in attentional performance across mindfulness interventions.

A number of correlational studies have examined the associations among dispositional measures of mindfulness and various facets of attentional control in older adults. Across studies, older adults show higher levels of self-reported dispositional mindfulness compared with young adults (Frank et al., 2015; Prakash et al., 2015; Fountain-Zaragoza et al., 2016). However, existing cross-sectional studies have found non-significant association between mindfulness, working memory, and inhibitory control (Prakash et al., 2015) and both positive (Fiocco and Mallya, 2015) and non-significant (Prakash et al., 2015) associations between this trait and set-shifting. Notably, despite Fiocco and Mallya's finding of a positive association with set shifting, neither study found a significant association with cost of shifting. One important methodological difference that could be contributing to the discrepant findings between these two studies is that the study by Fiocco and Mallya (2015) employed a paper and pencil measure (Trail Making Task B) that yields a total time to complete a 25-item task, whereas the study by Prakash et al. (2015) employed a computerized paradigm that allows for an examination of both accuracy and reaction time. Future investigations should continue to employ theoretically-informed measures and might benefit from the use of computerized paradigms that provide more detailed indices of performance.

A cross-sectional comparison of older adults with at least 10 years of meditation experience to age-matched individuals with no experience found that meditators exhibited better inhibitory control, processing speed, set-shifting, and working memory (Prakash et al., 2012a). Another study comparing older-adult meditators with a much broader range of meditation experience (1–29 years) to age-matched naïve controls and young-adult naïve controls revealed a smaller attentional blink in meditators (van Leeuwen et al., 2009). This finding suggests that older-adult meditators exhibited attentional blink benefits not only compared to their age-group peers but to younger individuals who have not experienced age-related attentional decline. Although these comparison studies provide information about the benefits associated with long-term practice, they are inherently limited by their cross-sectional nature. Instead, the use of randomized controlled trials (RCTs) of mindfulness training provides the most concrete evidence for benefits that may be directly attributed to mindfulness training.

One of the seminal studies to examine the causal role of mindfulness training in improving cognitive functioning of older adults compared transcendental meditation, involving the use of a mantra as a tool for turning attention inward to subtler levels of thought with: (1) the Langer mindfulness training method, in which the emphasis is placed on creative ways of problem solving; (2) a mental relaxation group; and (3) a no treatment condition. Participants were assessed on measures of paired associate learning, cognitive flexibility, and word fluency (Alexander et al., 1989). They found that the transcendental meditation group improved more than the mindfulness group, but that both were superior to relaxation and no treatment, on paired associate learning and cognitive flexibility, and mindfulness and TM improved similarly on word fluency. These results provided preliminary evidence for cognitive benefits following transcendental meditation, which has direct parallels with the current mindfulness training approaches.

More recently, three RCTs have evaluated the effects of the more standardized and widely used 8-week Mindfulness Based Stress Reduction program (MBSR; Kabat-Zinn, 1990) on cognitive outcomes in older adults. One study collected data on Trial Making Tests A and B, calculating a Trails B/A ratio as an index of executive function adjusted for processing speed (Moynihan et al., 2013). This study found that participation in the MBSR program significantly reduced the Trails B/A ratio immediately following the intervention compared to a waitlist condition; however, this difference was not maintained when assessed 3 and 24 weeks post-intervention (Moynihan et al., 2013). Another study found no differences in Trail Making Tests A and B, or a verbal fluency task that also indexes executive function, in MBSR compared to a reading and relaxation comparison group (Mallya and Fiocco, 2016). Thus, it is possible that the benefits observed by Moynihan et al. (2013) were not attributable solely to mindfulness training, but instead resulted from nonspecific factors as they did not include an active comparison condition. These might include interacting with others in a group setting and engaging in at-home practice of any sort, demand characteristics or expectancy effects arising from participation in an intervention study targeting attentional abilities, and practice effects resulting from repeated assessment. The third study evaluated the effect of an 8-week MBSR program, as well as the feasibility of a 12-week extended program, on immediate and delayed verbal memory, verbal fluency, inhibitory control, and working memory in older adults with clinically significant anxiety and worry symptoms (Lenze et al., 2014). This study did not employ a control group. Following the 8-week MBSR program, participants exhibited improved performance on all cognitive measures with the exception of immediate list learning, with no significantly superior outcomes for the 12-week program. Of note, one additional study evaluated Mindfulness Based Cognitive Therapy (MBCT), a clinical group intervention that involves elements of both cognitive-behavioral therapy and mindfulness meditation training (Segal et al., 2002), for bereaved older adults. This study found no significant improvements in attentional control, measured via working memory, compared to a waitlist comparison group (O'Connor et al., 2014). This lack of observed effects might be attributable to the clinical nature of the sample as depressive symptoms are associated with cognitive deficits (see Austin et al., 2001 for review). Additionally, it is again the case that the attentional improvements observed by Lenze et al. (2014) may have been due to nonspecific factors as these effects disappeared when a control group was included in the study by O'Connor et al. (2014).

Given the relative dearth of RCT studies in the literature, there is a clear need for further rigorous investigation of the attentional benefits following mindfulness training in older adults (See Future Directions box in Figure 1). Considering the many facets of attentional control and the various measures that can be employed to assess such abilities, it will be important for the field to take a systematic approach to the evaluation of the benefits of mindfulness. Future studies should adopt a framework through which they can base their conceptualization and measurement of attentional control in order to conduct hypothesis-driven experiments. First, adopting a framework based on the work of Dr. Raja Parasuraman and his colleagues would provide researchers with well-defined attention variables that exhibit well-characterized age differences. The use of such theoretically informed dependent variables would allow for an examination the degree to which mindfulness training alters (1) attentional processes that are driven by internally-mediated task-sets through the use of vigilance tasks to examine sustained attention and inhibitory control and (2) attention to externally based stimulus properties through the use of cued-visual search tasks to examine the shifting and scaling of perceptual attention. Second, mind-wandering, or the direction of attention away from the task at hand and toward task-irrelevant information (Smallwood and Schooler, 2006), represents a potential mechanism through which mindfulness might impact attention. Interestingly, although mind-wandering decreases markedly with age (Giambra, 1989; Jackson and Balota, 2012), there is preliminary evidence that older adults who are higher in trait mindfulness exhibit the least amount of task-unrelated mind-wandering (Fountain-Zaragoza et al., 2016). Further, we have found that older adults who participated in 4 weeks of mindfulness training exhibited significantly decreased task-unrelated mind-wandering during a cognitive task compared to an active control group (Whitmoyer et al., under review). Thus, future studies might examine the degree to which mindfulness training further accentuates the decrease in mind-wandering observed across age, and whether this provides compensatory benefits for attentional performance.

By building from a systematic, basic science foundation, researchers can best choose and measure meaningful outcome variables for intervention studies. It will also be important for studies to evaluate specific mindfulness training programs in order to provide aggregate evidence for a particular program's benefits. This will necessitate the creation and use of standardized manuals for the implementation of such training programs. Studies should have randomized designs and include active comparison groups, in which control participants engage in training that is devoid of the component of interest (i.e., mindfulness) but that is matched for key nonspecific factors (e.g., duration, group size, instructor expertise, etc.), in addition to waitlist control groups that receive no training. Such practices will allow us to characterize the benefits that are specific to mindfulness training, rather than group interventions in general, and bolster our ability to make causal claims. And finally, much is still unknown regarding the dose-response relationship between mindfulness training and attentional improvements, the longitudinal impacts of training, and the degree of transfer of benefits to other domains of cognitive functioning in older adults. Future studies incorporating more ecologically valid measures of cognitive and affective functioning can allow for a systematic examination of the benefits of mindfulness training for older adults.

## Mindfulness and neural correlates of attentional control

### Age-related alterations in neural activity and connectivity

Much work has focused on characterizing the neural changes that occur across development and elucidating the contribution of such changes to cognitive decline. In comparison to the more selective recruitment of the right prefrontal cortex observed in young adults during tasks of attentional control, older adults showed decreased prefrontal lateralization (Cabeza, 2002), over-recruitment of attentional control regions (Cabeza et al., 2004; Langenecker et al., 2004; Colcombe et al., 2005), and a decreased ability to modulate recruitment of attentional control regions in response to increasing demand (Prakash et al., 2009, 2012c). Although some studies have provided evidence for a positive association between activation of attentional control regions and behavioral performance in older adults (Reuter-Lorenz et al., 2000; Cabeza et al., 2004), more recent longitudinal data suggested that increases in frontal activation were associated with declines in performance for tasks of abstraction, chunking, inhibition, discrimination, switching, and manipulation (Goh et al., 2012). In addition, there is evidence of an age-related decrease in suppression of the default-mode network (DMN) which is activated during rest and internal, self-referential thought (Raichle et al., 2001; Raichle and Snyder, 2007; Buckner et al., 2008). The DMN is anticorrelated with executive control network activity during attentional control tasks (Fox et al., 2005) and the degree of this anticorrelation is associated with cognitive performance (Kelly et al., 2008). The importance of the DMN in cognitive aging is further highlighted by evidence that functional connectivity within the DMN (Andrews-Hanna et al., 2007; Damoiseaux et al., 2008; Koch et al., 2010; Voss et al., 2010), as well as the cingulo-opercular and frontoparietal control networks (Geerligs et al., 2015), decreases with age. During cognitive tasks, older adults exhibited less suppression of DMN regions compared to younger adults (Lustig et al., 2003), particularly in response to increasing task demands (Prakash et al., 2009; Sambataro et al., 2010), which was associated with poorer performance (Prakash et al., 2012c).

### Mindfulness and neural functioning

Theoretical accounts of mindfulness posit that its salutary effects on attentional and emotional regulation occur through increased top-down modulation of limbic and brainstem systems by the prefrontal cortex (Chiesa et al., 2013; Prakash et al., 2014). This model has been substantiated by evidence of improved resource allocation during early processing (Malinowski, 2013) and increased recruitment of attentional control regions, such as the prefrontal cortex and anterior cingulate cortex, in those who have received meditation training (see Chiesa and Serretti, 2009; Hölzel et al., 2011; Tang et al., 2015 for review). Notably, these are some of the same regions that have been identified as showing alterations in function with age that are implicated in age-related changes in cognitive performance.

Given that mindfulness involves the active allocation of attention, either to internal or external stimuli, trait levels of mindfulness are hypothesized to be associated with preserved integrity of the DMN with advanced age. A cross-sectional investigation testing this hypothesis found that mindfulness disposition in older adults was in fact associated with greater integrity of the DMN, particularly in the dorsal posterior cingulate cortex and precuneus (Prakash et al., 2012b). These regions are considered to be important hubs within the functional connectome that are highly implicated in integrating and processing information (Buckner et al., 2009). Specifically, the dorsal posterior cingulate serves as an interface between the DMN and the task-positive attentional control network (Leech et al., 2011), a role that is critical for efficient cognitive functioning. Another cross-sectional study, comparing adult expert meditators (mean age = 37) to individuals with no prior meditation experience (mean age = 36), found that naïve controls exhibited the expected negative correlations between age and total gray matter volume as well as age and attentional performance, whereas these associations were not observed in expert meditators (Pagnoni and Cekic, 2007). However, it is unclear what maximum age was included in this study, potentially limiting its applicability to elderly individuals. Together, these preliminary data suggest a potential role of mindfulness in preserving brain integrity with age, but they are limited by their cross-sectional nature.

The examination of neural outcomes as a function of mindfulness training in older adults is currently limited to one study. This RCT examined the effects of an 8-week MBSR program on neural activation in older adults (Moynihan et al., 2013). Following mindfulness training, improvements in executive control, indexed as the Trails B/A ratio, were found to be accompanied by a reduced shift toward rightward frontal alpha activation, which is associated with avoidance or withdrawal, but rather sustained left frontal alpha activation, which is associated with appetitive approach behaviors. These findings were similar to an RCT conducted in young to middle-aged adults (23–56 years) that found increases in left-sided anterior activation following 8-week MBSR, a neural pattern that has been associated with positive affect (Davidson et al., 2003b).

The reviewed studies provide preliminary evidence that mindfulness training might have implications for preventing and/or ameliorating age-related declines in brain structure and function and associated cognitive functions. However, much work is needed to more fully characterize the benefits of mindfulness training for neural functioning. One unique challenge presented by the study of older adults is the large variability in structural and functional changes in the brain with age (e.g., Raz et al., 2010). This heterogeneity is not accounted for by commonly used group-based statistical approaches, thus obscuring our understanding of age-related differences in attention as well as group-based changes following mindfulness training interventions. Longitudinal studies examining within-individual changes in neural variables provide detailed information regarding the trajectories of change across age (e.g., Goh et al., 2012, 2013). However, researchers will need to be innovative in their study design and use of neuroimaging techniques to account for both individual and group-based differences when examining the effects of mindfulness interventions. Initial investigations in this field should attempt to recruit relatively homogenous samples of healthy older-adults in an effort to control for comorbid conditions (e.g., psychiatric, autoimmune, neurodegenerative, etc.) and lifestyle factors that might impact neural integrity and associated attentional functions. Further, implementation of randomized, pre-post study designs with comparison to both active and waitlist control groups will provide the most accurate depictions of neural change resulting from mindfulness training.

## Mindfulness and psychological well-being

Successful aging is not limited to preserved cognitive function, but is conceptualized as multi-dimensional, including the preservation of both physical and cognitive functions, the maintenance of social interactions, and continued engagement in meaningful activities (Rowe and Kahn, 1997). Critically, attentional control is implicated in many of these aspects of older adults' daily functioning. For example, executive function is associated with functional status, as measured using the instrumental activities of daily living scale (Cahn-Weiner et al., 2000; Royall et al., 2004), as well as medical comprehension, decision-making, and adherence (Park, 1999; Insel et al., 2006). Such outcomes are important given that the percentage of adults with multimorbidity, or multiple chronic health conditions, increases significantly with age, and these conditions are associated with greater risks of disability, poor functional status, and poor quality of life (Salive, 2013).

Quality of life and well-being are integrally important to the holistic health of older adults, particularly as isolation increases due to decreases in the number and frequency of social contacts (Steptoe et al., 2013). Social isolation is associated with heightened inflammatory responses and greater risk for morbidity, which is associated with greater risks of poor functional status and poor quality of life (Salive, 2013). Mindfulness training is uniquely relevant to older adults who are at increased risk of experiencing chronic disease and pain in that internal sensations, both cognitive and physical, are often a central focus of training programs. Further, we have argued that mindfulness training is especially pertinent to older adults given its emphasis on emotion regulation, an ability that is highly preserved across age (Prakash et al., 2014). Central to socioemotional selectivity theory (Carstensen, 1993, 2006; Carstensen et al., 1999; Reed and Carstensen, 2012) is the finding that older adults exhibit increased prioritization of emotional goals and improvements in emotion regulation abilities with age. We have proposed that mindfulness training capitalizes upon this shift in motivation away from future-oriented goals toward present-focused optimization of emotional experience observed in older adults (Prakash et al., 2014). Given the interdependence of emotional and attentional control processes, mindfulness training simultaneously optimizes the use of attentional control abilities and the enactment of useful emotion regulation strategies.

Preliminary cross-sectional evidence suggested that trait levels of mindfulness were associated with enhanced psychological well-being, measured as self-reported depressive symptoms, quality of life, and stress, in older adults (Fiocco and Mallya, 2015) and that emotion regulation mediated the relationship between trait mindfulness and reduced perceived stress in both older and younger adults (Prakash et al., 2015). A detailed examination of emotion regulation strategy use during idiographic situations revealed that older adults reported greater use of acceptance-based strategies and less use of maladaptive strategies than young adults in moderate-intensity situations and situations evoking anxiety and sadness as well as less use of maladaptive strategies in high-intensity situations (Schirda et al., 2016). Additionally, reported use of thought avoidance mediated the association between mindfulness and emotion dysregulation and this effect was dependent on age such that less mindfulness in young, but not older, adults was associated with greater use of thought avoidance and greater emotion dysregulation (Prakash et al., 2017). Further, both qualitative and experimental research provide encouraging evidence of emotional benefits following mindfulness training.

Qualitative studies have used focus groups and content analysis of group discussions and diaries to explore the central themes reported by individuals who have participated in mindfulness training. One such study in older adults suffering from chronic pain found that participants reported beneficial effects of mindfulness training on pain, sleep, and achieving well-being (Morone et al., 2008). Participants reported that enhanced well-being was reflected in both elevated mood and increased global quality of life. Similarly, older adults with clinically significant depression or anxiety who participated in MBCT reported improvements in anxiety, depression, ruminative thoughts, and decreases in sleep-related problems (Foulk et al., 2014). Key topics discussed by a group of older-adult, low-income, African American women who participated in an MBSR program included the social support they received during group meditation, the use of mindfulness for stress management, and the application of mindfulness in their daily lives (Szanton et al., 2011). These participants reported using mindfulness skills to cope with a variety of stressors including managing depression and anger; growing older and having physical pain; medical tests; financial strain; as well as having grandchildren with significant mental, physical, financial, or legal hardships. These highlight the positive experiences and many perceived benefits participants freely reported following various forms of mindfulness training. Although this information is useful in determining the acceptability and feasibility of such programs, the lack of quantitative results limits the interpretation of statistical and clinical significance of mindfulness training's effects.

Expanding upon these qualitative data, other investigations of mindfulness training have provided experimental evidence for mindfulness training's ability to ameliorate emotional distress and promote well-being. For example, an 8-week MBSR program produced significant reductions in loneliness compared to a waitlist group (Creswell et al., 2012). In a sample of older adults reporting stress or symptoms of depression or anxiety, MBCT had a moderate to large effect on increasing trait mindfulness levels and improving emotional well-being, which were positively associated with one another (Splevins et al., 2009). However, participants in this study were not randomly assigned to MBCT and there was no control group with which to compare the effects. When comparing mindfulness training to an education control group for older adults with lower back pain, no between-group differences were observed on several outcomes (Morone et al., 2009). Instead, both groups exhibited significant improvements on measures of quality of life, self-efficacy, disability, and pain at post-intervention, which were maintained at 4-month follow-up. As was discussed in the previous sections of this review, these findings again suggest that factors not specific to mindfulness may have affected outcomes. Particularly, the qualitative studies described above point to the potential benefit of receiving social support for improving multiple domains of well-being, which is not a mindfulness-specific component of training. These effects are undetectable when mindfulness training is not tested against an active comparison condition. Thus, rigorous experimental investigation must be pursued in order to replicate previous findings and further characterize the benefits of mindfulness training.

The use of mindfulness training in clinical settings is of great interest, and there is accumulating evidence that such interventions can be useful in reducing symptoms of psychopathology. MBSR has been found to produce a >50% reduction in the number of older-adult participants with clinically significant depression and anxiety (Young and Baime, 2010). In older adults reporting worry symptoms with co-occurring cognitive dysfunction, there was a large effect size for increased mindfulness and reduced worry severity following MBSR, although no control group was used for comparison in this study (Lenze et al., 2014). These findings have been corroborated by meta-analytic evidence in young to middle aged adults with chronic medical diseases. This meta-analysis found that MBSR produces small, significant effects on psychological distress as well as psychopathology symptoms, including depression and anxiety (Bohlmeijer et al., 2010). Interestingly, the study by Splevins et al. (2009) suggests that specific components of mindfulness might confer greater psychological benefits than others. They found that the “act with awareness” and “accept without judgment” facets were associated with greater reductions in depression symptoms, whereas the other facets (observe and describe) were not.

Together, the results of studies evaluating the emotional benefits of mindfulness are promising. Mindfulness training appears to yield benefits for older adults with both clinical and sub-clinical symptoms of emotional distress, highlighting the potential for flexible application of mindfulness in many contexts. Moreover, the effects of mindfulness are not limited to reducing negative symptoms, such as depression and anxiety, but extend into increasing social support and promoting well-being. Nonetheless, there is still much to be learned regarding the effect of mindfulness-specific components on psychological health for both community-dwelling and clinical populations of older adults.

## Mindfulness and inflammatory processes

Symptoms of emotional distress and/or psychopathology can be accompanied by changes in inflammatory processes, which are further linked to a myriad of health sequelae. Although much is still unknown regarding the full spectrum of mindfulness training's health benefits, there is a great deal of interest in using mindfulness training programs to improve health both in clinical and community-dwelling settings. There are several mechanisms through which mindfulness training may alter inflammatory processes: alteration of hypothalamic-pituitary-adrenal axis or sympathetic nervous system functioning. These two systems are implicated in the transduction of the brain's perception of socio-environmental conditions into genomic responses through their production of stress-related hormones such as cortisol, epinephrine, and norepinephrine that directly alter expression of pro-inflammatory genes (Cole, 2009).

In a study of community-dwelling older adults, baseline levels of loneliness were associated with expression of the pro-inflammatory gene NF-κB in leukocytes and those who participated in an 8-week MBSR course exhibited significant down-regulation of NF-κB expression compared to a waitlist group (Creswell et al., 2012). However, the impact of mindfulness training on protein indicators of activated inflammatory responses was mixed. Although it was hypothesized that the MBSR group would exhibit reductions in all protein indicators, there was a significant reduction in serum C-reactive protein levels, but no significant change in IL-6 protein levels. A subsequent study attempted to parcel out the differential effects of specific components of MBSR on inflammatory processes in comparison to a waitlist group (Gallegos et al., 2013). This study likewise found no effects on IL-6 levels across groups. However, yoga practice and sitting meditation were associated with higher post-treatment insulin-like growth factor (IGF-1) concentrations, a protein that is implicated in neurogenesis and preserved cognitive function but that decreases with age (Anderson et al., 2002), and yoga practice was associated with greater improvement in positive affect across the intervention. However, body scanning practice, in which focused attention is directed to the sensations of successive areas of the body, was found to have negative effects as it was associated with reduced antibody responses (IgM and IgG) at 3 weeks post-intervention; although this effect did not remain at 24 weeks. In contrast, another study found the expected increase in IgG response following 8-week MBSR compared to waitlist, but this effect disappeared at 24 weeks post-intervention. It is important to note that these studies are all limited by the lack of active comparison groups with which to compare these changes.

The last study evaluating inflammatory processes in older adults recruited participants with moderate sleep disturbance who were randomized to a 6-week mindful awareness practices intervention or sleep hygiene education group (Black et al., 2015). Although those who participated in mindfulness training improved more on insomnia symptoms, depression symptoms, fatigue interference, and fatigue severity, there were no between-group differences for anxiety, stress, or NF-κB expression. Instead, NF-κB expression was significantly down-regulated over time in both groups. This finding highlights the sizable influences that can result from non-specific factors arising from participation in group-based interventions. It is critical that future RCTs utilize active control conditions to clarify the impact of mindfulness training on inflammatory processes above and beyond the benefits of social engagement that participants might garner from any type of training. In addition to elucidating mindfulness-specific effects on inflammatory processes, future studies must evaluate the degree to which these effects persist over time and have broader systemic benefits.

## Conclusions and future directions

There is great interest, both from the public and from healthcare providers, in the application of mindfulness techniques. This review article discussed what is currently known about the effect of mindfulness training on key areas of interest within geropsychology: attentional control performance (behavioral and neural correlates), psychological well-being, and inflammatory processes. Although the majority of the reviewed studies provide positive results for mindfulness training in each of these domains, the field is currently limited in its scope and much more work is needed in order to establish the causal impact of mindfulness practice on these outcomes. Moreover, the conclusions that might be drawn from the existing studies are obscured by the heterogeneity of samples and limitations of the methods being employed. Whereas some studies focused on older adults with chronic health conditions, others recruited participants with specific psychological symptoms or diagnoses, and many aimed to examine relatively healthy older adults. There are also inconsistencies in the training programs being tested and a myriad of outcomes being evaluated within each domain. The creation and dissemination of standardized training protocols and identification of theoretically informed dependent variables will allow for the systematic evaluation of mindfulness training's effects. Further, given the relative dearth of RCTs, future studies will need to replicate existing findings and employ rigorous experimental tests in order to lay the foundation for the continued growth of this field.

Given that mindfulness is often broadly defined, and often considered to be multifaceted, future research should focus on identifying which components of mindfulness training confer which benefits. Developing and testing a mechanistic account of mindfulness training's effects will allow for the optimal application of training to promote healthy aging. Researchers might then begin to address the gaps in what is known about the degree to which these benefits are maintained longitudinally across continually advancing age. Another important target of future research is to examine to what extent these benefits transfer to broader functions that are critically implicated in the everyday lives of older adults. These might include comprehension of medical information, health behaviors, social engagement, and functional status, all of which have a foundation in intact attentional control processes.

The reviewed evidence suggests that mindfulness may be advantageous for promoting cognitive, emotional, and physical health within the context of advanced aging. Moreover, these beneficial effects are conferred to those with little to no psychological symptoms as well as those with diagnosed psychological or medical conditions. This suggests that mindfulness training might be easily integrated into a variety of contexts, such as senior centers and group homes, and that it would be valuable and appropriate for such heterogeneous audiences. We previously described an ideal training program for older adults as one that is pragmatic; that capitalizes on older adults' increased motivation toward emotional well-being; and that exhibits transfer effects to multiple domains ranging from specific cognitive processes to broad, everyday function (Prakash et al., 2014). In line with these criteria, we assert that mindfulness represents a potential intervention for not only reducing emotional distress in older adults, but for allowing them to flourish.

## Author contributions

SF and RP contributed significantly to the conception of the work. SF completed the initial drafting the work and RP provided critical revisions for important intellectual content. RP provided final approval of the version to be published. SF and RP agree to be accountable for all aspects of the work in ensuring that questions related to the accuracy or integrity of any part of the work are appropriately investigated and resolved.

### Conflict of interest statement

The authors declare that the research was conducted in the absence of any commercial or financial relationships that could be construed as a potential conflict of interest.
